# Identification of the *RcWRKY* family in *Rubus chingii* and analysis of its regulatory mechanisms for flavonoid synthesis

**DOI:** 10.3389/fpls.2025.1728584

**Published:** 2026-01-29

**Authors:** XiuRong Xu, Yan Cheng, Shiming Cheng

**Affiliations:** 1 Zhejiang Academy of Forestry, Hangzhou, Zhejiang, China; 2 Ningbo University of Finance and Economics, Ningbo, Zhejiang, China

**Keywords:** flavonoid, gene family, identification, *Rubus chingii*, WRKY

## Abstract

*Rubus chingii* Hu. is a versatile plant valued for its medicinal and edible properties. Its fruits are rich in flavonoids, with unripe fruits exhibiting higher flavonoid content. This study identified the *WRKY* gene family in this plant and explored its regulatory mechanisms in flavonoid synthesis. In total, 52 members of the *RcWRKY* family were identified; they were unevenly distributed on six chromosomes and all located in the nucleus. Based on the phylogenetic tree, these 52 members were divided into seven subgroups, and the motifs and gene structures of different subgroups were highly consistent. Their promoters were rich in elements such as light and jasmonic acid methyl response elements. Additionally, 20 collinear gene pairs were identified in the genome, most of which underwent purifying selection. Whole-genome duplication was the main cause of expansion of the *RcWRKY* family, and most members showed obvious collinearity with dicotyledonous plants. Transcriptome analysis revealed that 47 *RcWRKY* members were differentially expressed during fruit ripening, and 11 were highly expressed at the mature green (MG) stage with high flavonoid content. Additionally, the 30 identified flavonoid synthesis-related genes were highly expressed in the MG period, with six *RcWRKY* members being significantly positively correlated with most flavonoid synthesis genes. Reverse transcription-quantitative polymerase chain reaction and subcellular localization confirmed that *RcWRKY34* and *RcWRKY37* were highly expressed in the MG period and located in the nucleus. Dual-luciferase assay showed that both *RcWRKY34* and *RcWRKY37* positively regulated the flavonoid synthesis gene *LG07.48*. Overall, this study lays a foundation for enhancing the medicinal value of palm-leaved *R. chingii*.

## 1 Introduction

Flavonoids are a class of secondary metabolites that include flavonols, flavanols, anthocyanins, isoflavones, and other substances (Shen et al., 2022). They play important roles in the growth and development of plants and significantly impact human health, attracting increasing attention in various research fields in recent years (Li P. et al., 2021). The formation of plant flavonoids is not regulated by a single enzyme but by the combined effects of several enzymes (Deshmukh et al., 2018; Ni et al., 2020). Continuous development of sequencing technologies in recent years has increasingly clarified the flavonoid biosynthetic pathway. During the synthesis of plant flavonoids, phenylalanine is catalyzed by phenylalanine ammonia-lyase or phenylalanine deaminase (*PAL*) to form cinnamic acid, which is catalyzed by cinnamate 4-hydroxylase (*C4H*) and 4-coumarate coenzyme A ligase (*4CL*) to form cinnamoyl coenzyme A. Cinnamoyl coenzyme A is further catalyzed by chalcone synthase (*CHS*) to form chalcone, which is converted to hesperetin by chalcone isomerase (*CHI*). Hesperetin is catalyzed by flavonoid 3-hydroxylase (*F3H*) to form dihydrokaempferol, which is catalyzed by flavonoid 3’-hydroxylase (*F3’H*) to form dihydroquercetin. Dihydrokaempferol and dihydroquercetin are formed by flavonol synthase (*FLS*) or dihydroflavonol 4-reductase (*DFR*) (Du et al., 2024; Liu et al., 2022). The entire key pathway involves 10 gene family proteins, among which *PAL*, *4CL*, *CHS*, *CHI*, and *DFR* are independent gene family proteins, whereas *F3H*, *FLS*, and *ANS* belong to the 2-oxoglutarate-dependent dioxygenase or α-ketoglutarate-dependent dioxygenase supergene family proteins, and *C4H* and *F3’H* belong to the cytochrome P450 supergene family proteins (Du et al., 2024).

Recently, *WRKY* gene families have been identified in more species owing to the rapid development of genomics. For example, 89 *PsWRKY* genes have been identified in pea (Xiong et al., 2024), 82 *CtWRKY* genes in safflower (Song et al., 2023), 103 *CpWRKY* genes in cucumber (Liu et al., 2024), 57 *BpWRKY* genes in rockcress (Chen et al., 2025), and 55 *AhyWRKY* genes in grain Amaranth (Singh et al., 2025). Transcription factors play a significant role in regulating flavonoid synthesis in plants. For instance, transcription factors such as R2R3-MYB, bHLH, WD40, and WRKY regulate genes associated with flavonoid synthesis (Zheng et al., 2025) Recent studies have reported that *WRKY* plays an important role in regulating plant flavonoids. For example, *PeWRKY30* positively regulates, whereas *PeWRKY12* negatively regulates flavonoid synthesis in passion fruit (Ma et al., 2024). In grapes, *VvWRKY70* inhibits *VvCHS* expression, thereby inhibiting flavonoid synthesis (Wei et al., 2023). In apples, *MdWRKY50* promotes *MdCHS* synthesis, thereby enhancing flavonoid synthesis (Bai et al., 2024). In pears, *PbWRKY75* promotes *PbDFR* synthesis, thereby enhancing flavonoid synthesis (Cong et al., 2021). Interestingly, in addition to directly regulating the expression of genes related to flavonoid synthesis, WRKY transcription factors indirectly regulate flavonoid synthesis by modulating the expression of transcription factors such as R2R3-MYB, bHLH, and WD40. These reports suggest that WRKY plays an important role in regulating the secondary metabolism of plant flavonoids (Li et al., 2025).

*R. chingii* contains a large amount of flavonoid secondary metabolites, and WRKY transcription factors play an important role in regulating plant flavonoids (Hua et al., 2023; Chen et al., 2021; Zhu et al., 2024; Tian et al., 2024). Therefore, we investigated the expression patterns of *RcWRKY* genes in *R. chingii* fruits during ripening to identify the key WRKY transcription factors regulating flavonoid-related processes. Previous studies have enhanced our understanding of the functions of the *WRKY* gene family. Comparison of related studies on gene families indicates that, due to differences in the protein sequence structure, the *WRKY* family in plants is divided into seven subfamilies: Groups I, II-a–e, and III. Group I contains two WRKY domains, groups II-a–e contain WRKY domains and diverse C2H2-type zinc finger structures, and group III contains WRKY domains and C2HC-type zinc finger structures (Fan et al., 2025). Despite its identification in multiple species, the *WRKY* family has not yet been identified *R. chingii*, belonging to the *Rubus* genus in the *Rosaceae* family, is a multifunctional medicinal and edible plant (Xu et al., 2024). Its fruits contain abundant flavonoids, with unripe fruits showing higher flavonoid content, which may explain why traditional Chinese medicines often use unripe fruits (Chen et al., 2021; Hua et al., 2023; Shan et al., 2025). Unlike those of red and black raspberries, drupelets of *R. chingii* do not detach from the receptacle at maturity. Typical phases of fruit maturation are the mature green (MG), green yellow (GY), orange yellow (YO), and red (RE) phases. MG and GY fruits are both hard but of different colors, YO fruits begin to soften and become orange, and RE fruits rapidly soften and become red during maturation. Unripe fruits are traditionally used in Chinese medicine, whereas ripe fruits are preferred by consumers for their unique flavor and nutritional properties. To elucidate the regulatory mechanisms of flavonoid synthesis during fruit ripening, this study identified the *WRKY* gene family in *R. chingii*, conducted bioinformatics analysis of each member, identified the regulatory family members involved in flavonoid synthesis, and performed molecular biology verification, laying a foundation to further improve the medicinal value of *R. chingii* using molecular biological methods in the future.

## 2 Materials and methods

### 2.1 Identification of the *RcWRKY* family, analysis of protein physicochemical properties, and construction of a phylogenetic tree

Initial data preparation involved downloading the genome sequence and annotation file of *R. chingii* from the *Rosaceae* genome database (https://www.rosaceae.org/), obtaining the WRKY domain file with the Pfam database number PF00403 (http://pfam-legacy.xfam.org/), and downloading all 71 AtWRKY protein sequences from The *Arabidopsis* Information Resource (TAIR; https://www.arabidopsis.org/). Subsequently, WRKY domain and all AtWRKY sequences were compared using the “Simple HMM Search” and “Blast Several Sequences to a Big Database” programs of the TBtools software, and the intersection of the comparison results was uploaded to the InterProScan database (https://www.ebi.ac.uk/interpro/result/interprosca/). Finally, all members of the *RcWRKY* family were identified (Chen et al., 2023).Simple Hmm Search results determine members based on an E-value less than 1e-10. The parameter setting is an E-value less than 1e-5, and the blast program is executed.

To better understand the basic information of the family members, this study analyzed the key physicochemical properties, such as amino acid quantity, molecular weight, isoelectric point, and instability coefficient of RcWRKY, using the Expasy ProtParam online tool (https://web.expasy.org/protparam/). Position information of the family members was extracted using the “GXF Gene Position & Info. Extract” program of the TBtools software (Chen et al., 2023; Su et al., 2023). Finally, specific locations of the family member proteins in cells were predicted using the Bologna Unified Subcellular Component Annotator database (https://busca.biocomp.unibo.it/).

To better understand the grouping of the family members, we constructed a phylogenetic tree of AtWRKY and RcWRKY. We aligned all protein sequences using the MAFFT v7.471 program, constructed a phylogenetic tree using the MEGA7 software, and displayed the phylogenetic tree on the Chiplot website (https://www.chiplot.online/). Based on the classification of the 71 AtWRKY protein sequences in TAIR database (https://www.arabidopsis.org/), RcWRKY proteins were grouped together.

### 2.2 Motif and gene structure analyses of the *RcWRKY* family

MEME online tool (https://meme-suite.org/) was used to analyze the motifs in the RcWRKY protein sequence, resulting in the identification of 10 conserved motif sequences. These conserved motif sequences were uploaded to the InterProScan database (https://www.ebi.ac.uk/interpro/result/interprosca/) for functional annotation. Finally, Gene Structure View of the TBtools software was used to visualize the motifs, conserved domains, and gene structures (Chen et al., 2023).

### 2.3 Analysis of *cis*-regulatory elements in the *RcWRKY* family

Using the Fasta Extract program of the TBtools software, the 2000-bp promoter sequence preceding the ATG start codon of all *RcWRKY* genes was extracted. This sequence was uploaded to the PlantCARE database (http://bioinformatics.psb.ugent.be/webtools/plantcare/) for *cis*-regulatory element prediction, and the number of such elements was counted. Finally, the results were visualized as a heat map using the HeatMap program of the TBtools software (Chen et al., 2023).

### 2.4 Analysis of collinearity in the genome of the *RcWRKY* family

Next, collinearity of the *RcWRKY* family members was analyzed using the MCScanX software, and a collinearity map was drawn using the Advanced Circos function of the TBtools software. Replication types of the *RcWRKY* family were analyzed using DupGenfinder. Replication types were classified into five categories: Whole-genome (WGD), transposed, dispersed, tandem (TD), and proximal duplication (Chen et al., 2023; Qiao et al., 2019; Wang et al., 2012). The non-synonymous substitution rate/synonymous substitution rate (Ka/Ks) of gene pairs with different replication types was calculated using the Simple Ka/Ks Calculator of the TBtools software (Chen et al., 2023).

### 2.5 Correlation analysis of the *RcWRKY* family among different species

The genome and annotation files of maize and grape were downloaded from the Chinese National Genome Database (https://ngdc.cncb.ac.cn/), those of rice and *Arabidopsis thaliana* from the Ensembl Plants database (https://plants.ensembl.org/), and those of *Fragaria vesca*, *Malus domestica*, *Pyrus communis*, *Prunus persica*, *R. idaeus*, and *R. occidentalis* from the *Rosaceae* Genome Database (https://www.rosaceae.org/). MCScanX software was used to conduct collinearity analysis of the *RcWRKY* family members of different species. Finally, the results were visualized as a heat map using the HeatMap program of the TBtools software (Chen et al., 2023; Wang et al., 2012).

### 2.6 Expression patterns of the *RcWRKY* and flavonoid synthesis gene family members during fruit ripening

Transcriptome data of *R. chingii* at different stages (MG, GY, YO, and RE) were obtained from the National Center for Biotechnology Information (bioproject number: PRJNA671545). The data were generated using the Illumina sequencing platform, and clean reads obtained from the National Center for Biotechnology Information fastq files were aligned with the *R. chingii* genome using the Hisat2 v2.0.5 software. The reads of each gene were calculated using the FeatureCounts tool (http://subread.sourceforge.net/), and fragments per kilobase pair per million reads values were calculated based on the transcript length.

Members of the flavonoid synthesis gene family were identified using the same method described in section 1.1. The 10 flavonoid synthesis-related protein structure domain files with Pfam database numbers (**Supplementary Table 1**) and protein sequences of 23 genes in 10 flavonoid synthesis-related gene families of *A. thaliana* were downloaded from TAIR database (https://www.arabidopsis.org/). Then, 10 flavonoid biosynthetic gene family members in *R. chingii* were identified, and a phylogenetic tree was constructed. Expression levels of the *RcWRKY* and flavonoid synthesis gene family members were statistically analyzed, and the average values for each period were calculated. HeatMap program of the TBtools software was used to visualize the heat map (Chen et al., 2023).

### 2.7 Analysis of interactions between the *RcWRKY* and flavonoid synthesis gene family members

Correlations between members of the *RcWRKY* and flavonoid synthesis gene families during fruit development were analyzed using the Chiplot website (https://www.chiplot.online/), and a correlation heatmap was constructed (Xie et al., 2023). Simultaneously, W-box sites in the promoters of all flavonoid synthesis gene family members were analyzed, as described in section 1.3. Finally, the binding sites of promoters were drawn using the Simple BioSequence Viewer program of the TBtools software (Chen et al., 2023).

### 2.8 Reverse transcription-quantitative polymerase chain reaction verification, subcellular localization, and dual-luciferase reporter gene verification

In order to detect the expression of genes at different stages of fruit ripening, *R. chingii* fruits were harvested at 30 days (MG), 40 days (GY), 50 days (YO), and 60 days (RE) after flowering. The fruits were rapidly frozen with liquid nitrogen and stored at -80°C in the refrigerator. Each replicate consisted of a minimum of 10 fruits collected from 5 to 6 *R. chingii* trees, meticulously mixed to ensure equal distribution. A minimum of six biological replicates (≥60 fruits in total) were established for each stages of *R. chingii*, amounting to a total of 240 fruits collected for this study. RNA was extracted from young leaves using the Plant RNA Extraction Kit (Takara, Beijing, China), and RNA purity was determined using the NanoPhotometer spectrophotometer (IMPLEN, CA, USA). RNA was reverse-transcribed into cDNA using the PrimeScriptTM RT Reagent Kit with gDNA Eraser (Takara).

RT-qPCR analysis of 12 samples (MG, GY, YO, and RE; three replicates for each treatment, totaling 12 samples) was performed using the SYBR PrimeScript RT-PCR Kit (Takara) and ABI 7500 Real-Time PCR system (ABI 7500; Thermo Fisher, Singapore). Relative gene expression was calculated using the 2^-ΔΔCT^ method (Livak and Schmittgen, 2001). Fluorescent qPCR primers were designed using the Batch q-PCR Primer Design tool in the TBtools software. All primers are listed in **Supplementary Table 2**; actin was used as the internal reference gene in *R. chingii* (Chen et al., 2023). Finally, a bar chart was constructed using GraphPad Prism v.10.

Using the seamless cloning technique, open reading frame sequences of the removed stop codons of *RcWRKY34* and *RcWRKY37* were ligated into the pAN580 vector, and green fluorescent protein was connected at the N-terminus. These recombinant vectors were introduced into tobacco protoplasts via polyethylene glycol-mediated transformation. Green fluorescent protein fluorescence signals were observed under 470 nm excitation light using a laser confocal microscope to analyze the localization of RcWRKY34 and RcWRKY37 in cells (Yoo et al., 2007).

Open reading frame sequences of *RcWRKY34* and *RcWRKY37* were ligated into the pGreen II 62-SK effect vector, and the 2000-bp promoter sequence before the ATG start codon of *LG07.48* was connected to *LUC* at the upper end of the pGreen II 0800-LUC vector. Both recombinant vectors were transformed into the *Agrobacterium* GV3101 strain of root-knot fungus, and tobacco leaves were infected with *Agrobacterium*. Three leaves were injected from each tobacco plant, and each leaf was injected with 9 areas (1 mL of bacterial solution could cover 9 areas). At least 3 tobacco plants were injected in the same batch to ensure the reliability of the results. Infected tobacco was cultured in the dark for 24 h and transferred to a 25°C constant-temperature incubator under 16 h alternating light and dark conditions for two days. LUC fluorescence signals were observed using a chemiluminescence imaging system (Tanon 5200; Tanon, Shanghai, China). LUC and REN fluorescence activities were measured using a dual-luciferase reporter gene detection kit (DL101-01; Vazyme, Nanjing, China), and the relative LUC/REN ratio was calculated.

## 3 Results

### 3.1 Identification of the *RcWRKY* family, analysis of protein physicochemical properties, and construction of a phylogenetic tree

**Figure 1:**
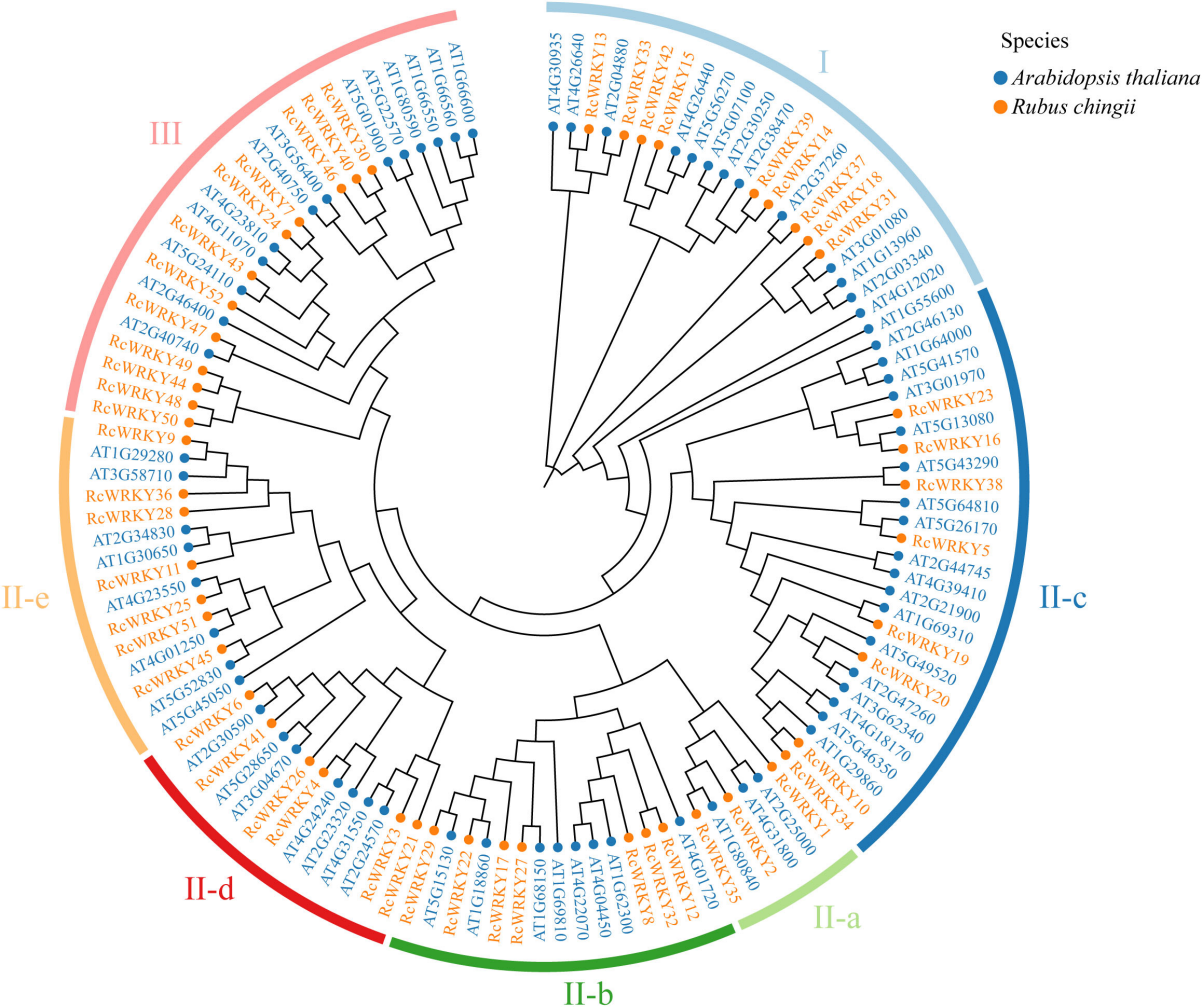
Phylogenetic tree based on the amino acid sequences of different species. At: *Arabidopsis thaliana*;Rc: *Rubus Chingii*.

In this study, we identified 52 *RcWRKY* family members in *R. chingii*. These 52 *RcWRKY* members were unevenly distributed across six chromosomes, with seven on chromosome 2, eight on chromosome 3, six on chromosome 4, seven on chromosome 5, 14 on chromosome 6, and 10 on chromosome 7 (**Supplementary Table 3**). Analysis of physicochemical properties revealed that the number of amino acids in these 52 proteins was 123–1448, molecular weight was 13520.87–159535 Da, isoelectric point was 4.53–9.96, and instability coefficient was 33.37–72.11, with an average hydrophilicity coefficient ranging from –1.189 to –0.354 (**Supplementary Table 3**). Subcellular localization prediction results showed that all 52 RcWRKY proteins were localized in the nucleus (**Supplementary Table 3**). By constructing a phylogenetic tree of WRKY proteins from *A. thaliana* and *R. chingii* and classifying them according to AtWRKY proteins in *A. thaliana*, this study divided RcWRKY proteins into groups I, II-a–e, and III. Group I contained nine RcWRKY and 14 AtWRKY proteins, group II-a contained three RcWRKY and three AtWRKY proteins, group II-b contained seven RcWRKY and eight AtWRKY proteins, group II-c contained eight RcWRKY and 18 AtWRKY proteins, group II-d contained six RcWRKY and seven AtWRKY proteins, group II-e contained seven RcWRKY and eight AtWRKY proteins, and group III contained 12 RcWRKY and 13 AtWRKY proteins (**Figure 1**).

### 3.2 Motif distribution and gene structure of the *RcWRKY* family

**Figure 2:**
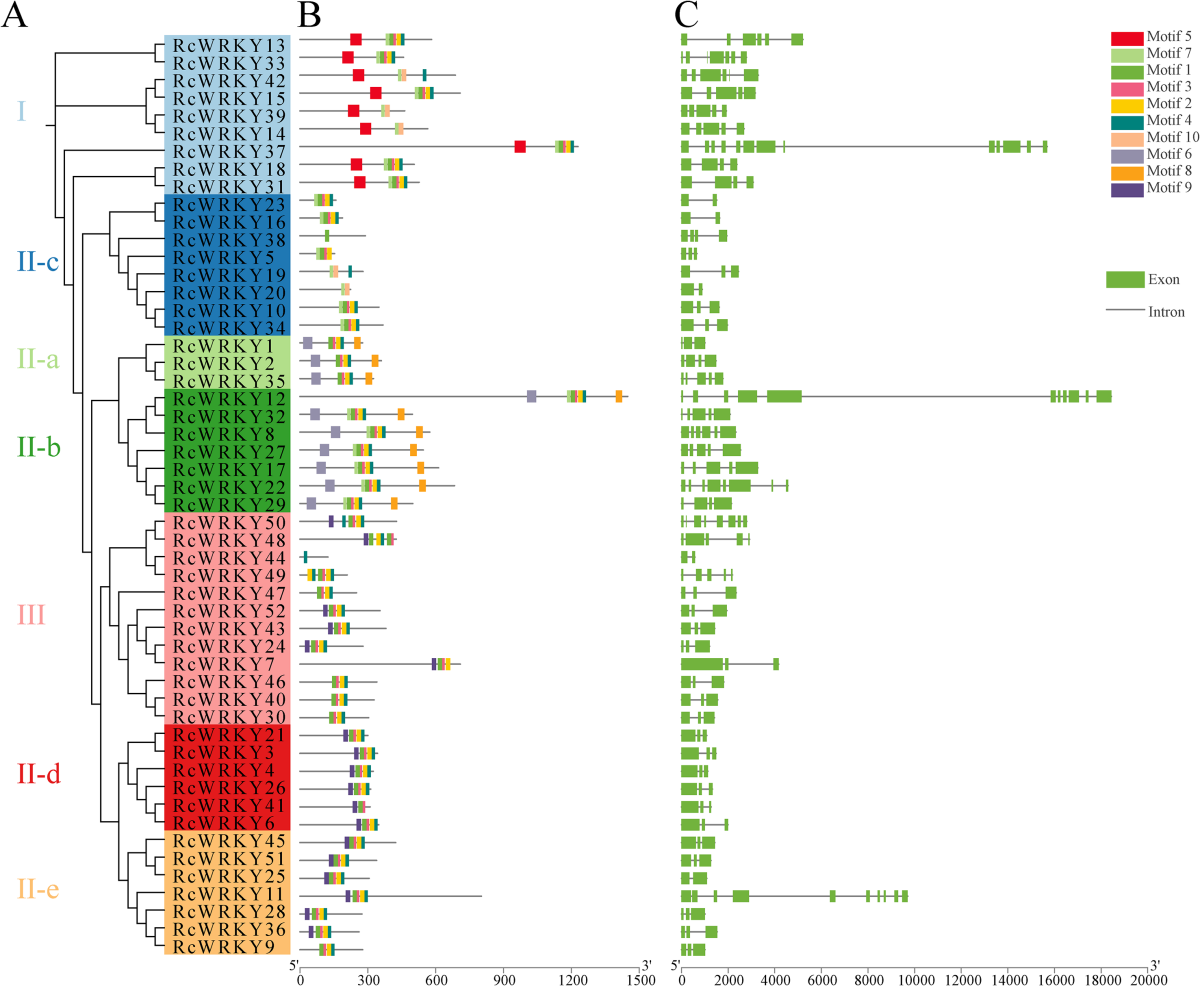
**(A)** Phylogenetic tree of the *RcWRKY* gene family; **(B)** Motif distribution of the *RcWRKY* gene family; **(C)** Structure of the *RcWRKY* gene family.

We assessed the distribution of the top 10 conserved motifs in the *RcWRKY* family. Notably, motif sequences in the gene family members of different groups exhibited a high degree of consistency, with some motifs being specific to certain groups, such as motif 5, which was present only in group I, and motif 6, which was present only in groups II-a and II-b (**Figures 2A, B**). Additionally, motifs 1/2/5/6/10 were conserved in the WRKY domain (**Supplementary Table 4**). Further analysis of the gene structures of the 52 *RcWRKY* family members revealed that the gene structures of members within the same group were highly consistent. For example, members of group II-c had 2–4 exons, whereas those of group II-d all had three exons (**Figure 2C**).

### 3.3 Number of *cis*-acting elements in the *RcWRKY* family

**Figure 3:**
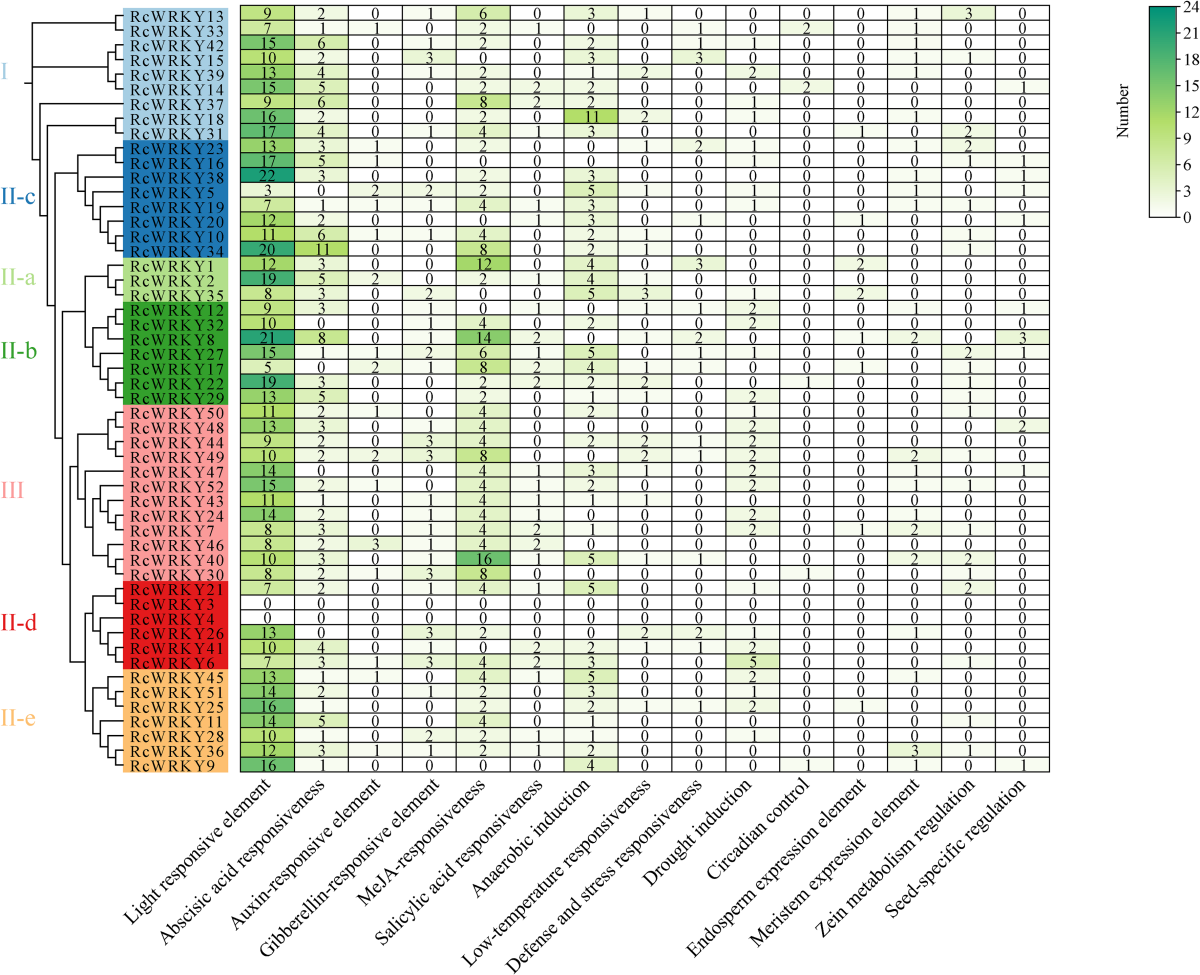
Statistical analysis of the number of *cis-*regulatory elements in the *RcWRKY* gene family.

Promoters of the *RcWRKY* family members were rich in various *cis*-acting elements, including response elements related to growth, development, hormones, and stress (**Figure 3**). Among these elements, those related to growth and development, especially photoreceptor elements, were the most abundant. The most common hormone response elements were abscisic acid and jasmonic acid methyl ester response elements. The most abundant stress response elements were anaerobic induction and drought response elements. The heatmap clearly showed that, except photoreceptor elements, the most abundant *cis*-acting elements in the *RcWRKY* family were jasmonic acid methyl ester response elements.

### 3.4 Analyses of collinearity and gene replication types in the *RcWRKY* family

**Figure 4:**
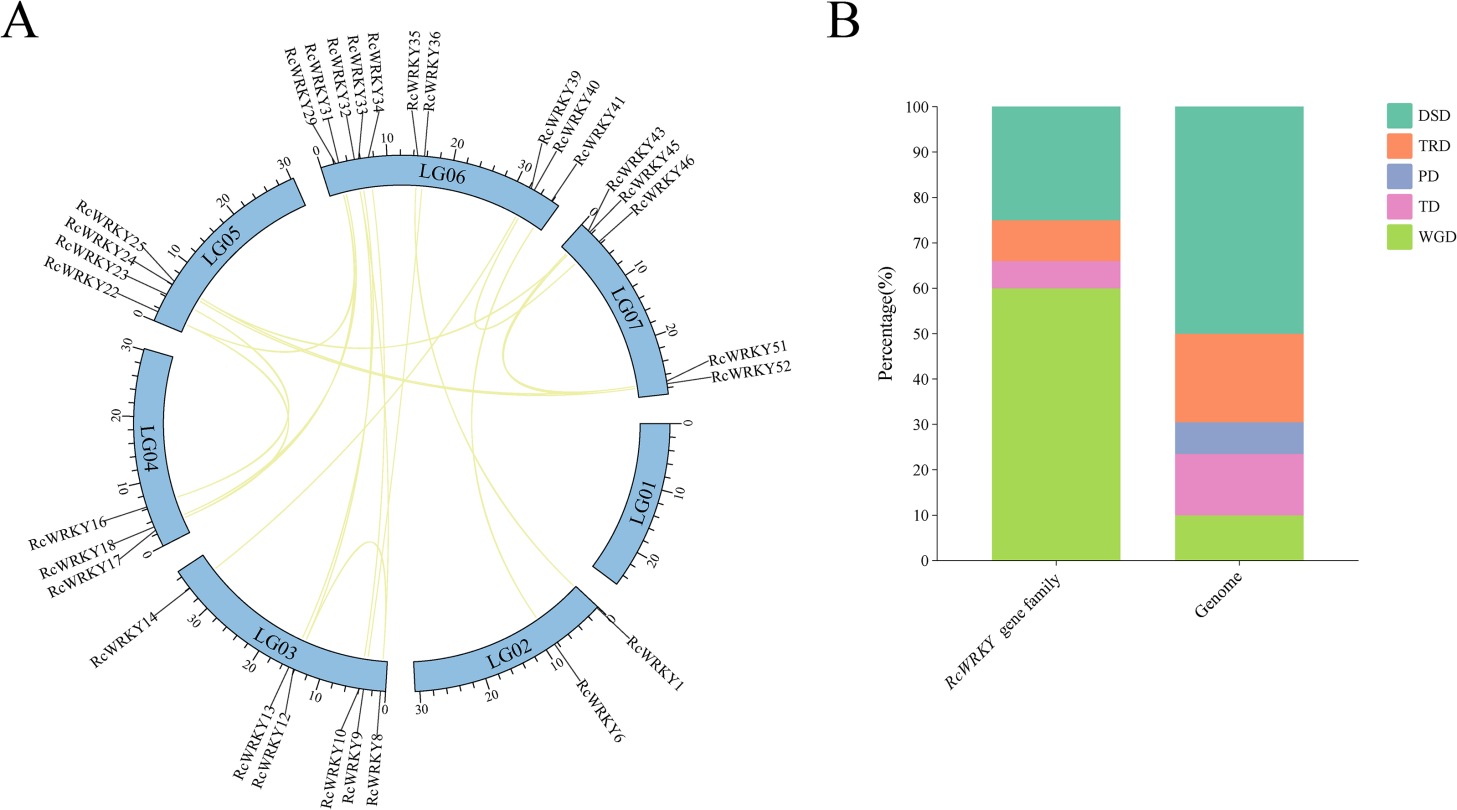
**(A)** Linear diagram of the *RcWRKY* gene family, **(B)** The proportion of the *RcWRKY* gene family in the overall genome replication pattern.

Collinearity analysis was conducted within the *R. chingii* genome, and 20 collinear gene pairs were identified (**Figure 4A**). Calculations of their Ka/Ks ratio revealed that, except for three gene pairs showing high divergence (*RcWRKY1*/*RcWRKY35*, *RcWRKY9*/*RcWRKY36*, and *RcWRKY25*/*RcWRKY45*), Ka/Ks ratios of other gene pairs were all less than 1 (**Supplementary Table 5**).The three gene pairs might be due to a low sequence similarity (such as a nucleotide similarity of less than 50%), and it is considered to be excluded from the analysis because the Ka/Ks ratio might be unreliable. Further analysis revealed eight gene pairs of dispersed duplication, three gene pairs of transposed duplication, two gene pairs of TD, and 20 gene pairs of WGD in the *RcWRKY* family (**Supplementary Table 6**). Compared to its role in the entire genome, WGD played a much greater role in *RcWRKY* family expansion (**Figure 4B**).

### 3.5 Homology of the *RcWRKY* family with other species

**Figure 5:**
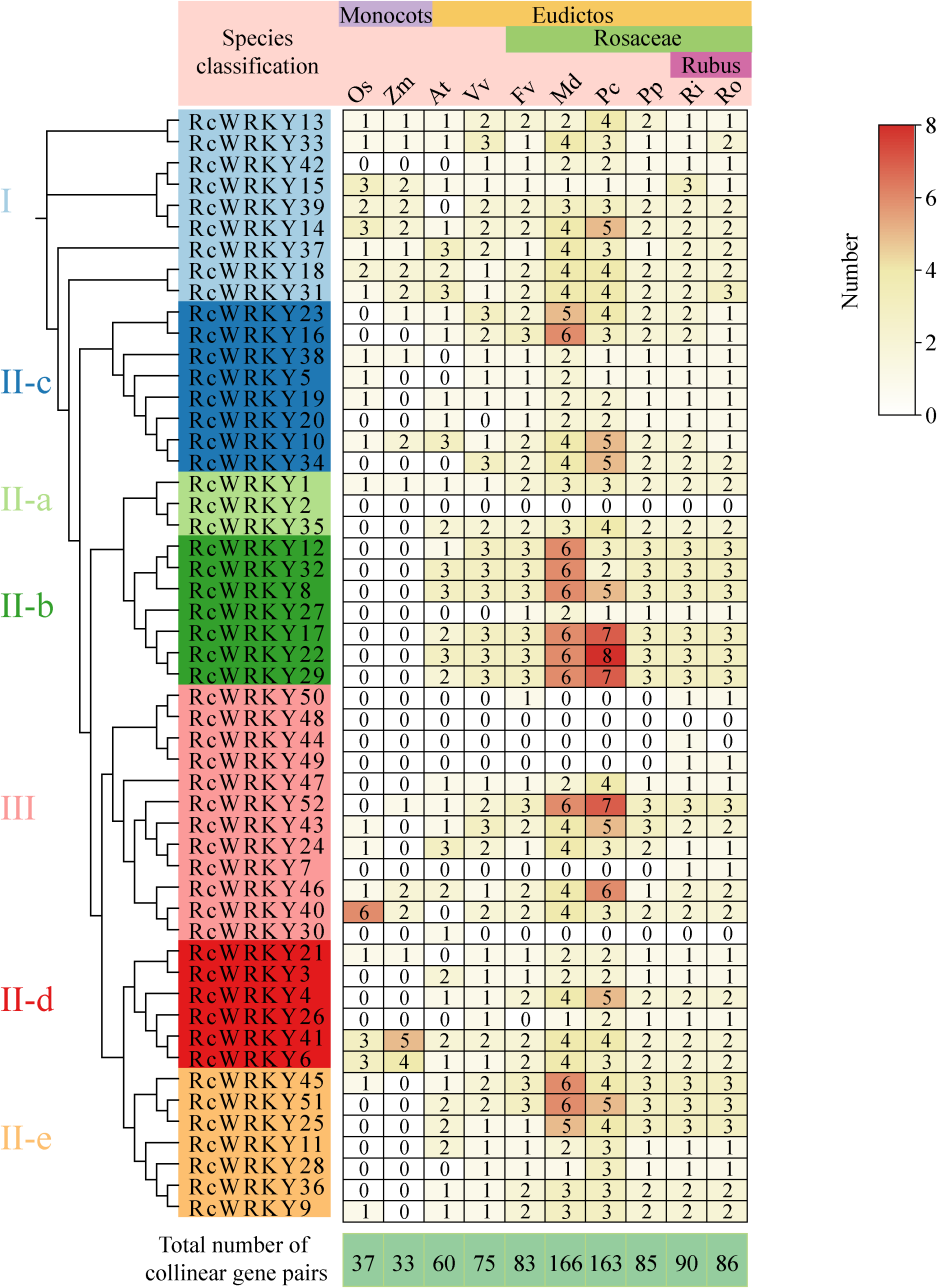
Statistical analysis of the number of orthologous gene pairs in the *RcWRKY* gene family compared to other species.(Os: Oryza sativa;Zm: Zea mays;At: *Arabidopsis thaliana*;Vv: Vitis vinifera;Fv: *Fragaria vesc*;Md: *Malus domestica*;pc:*Pyrus communis*;pp:*Prunus persica*;Ri: *Rubus Idaeus*;Ro: *Rubus occidentalis*;Among them, Oryza sativa and Zea mays are monocots plants, while the others are eudictos plants. Among the eudictos plants, except for *Arabidopsis* and grape, the rest belong to the Rosaceae family.).

Co-linearity analysis of the *RcWRKY* family members with other species revealed 37 collinear gene pairs with rice, 33 pairs with corn, 60 pairs with *A. thaliana*, 75 pairs with grape, 83 pairs with *F. vesca*, 166 pairs with *M. domestica*, 163 pairs with *Pyrus communis*, 85 pairs with *Prunus persica*, 90 pairs with *R. Idaeus*, and 86 pairs with *R. occidentalis* (**Figure 5**). Further analysis revealed that only a few *RcWRKY* members were collinear with monocotyledonous plants, whereas most were collinear with dicotyledonous plants. For example, members of group II-b were collinear only with dicotyledonous plants. Notably, *RcWRKY2* and *RcWRKY48* did not exhibit any collinear genes in other species (**Figure 5**).

### 3.6 Expression patterns of the *RcWRKY* family during fruit maturation

**Figure 6:**
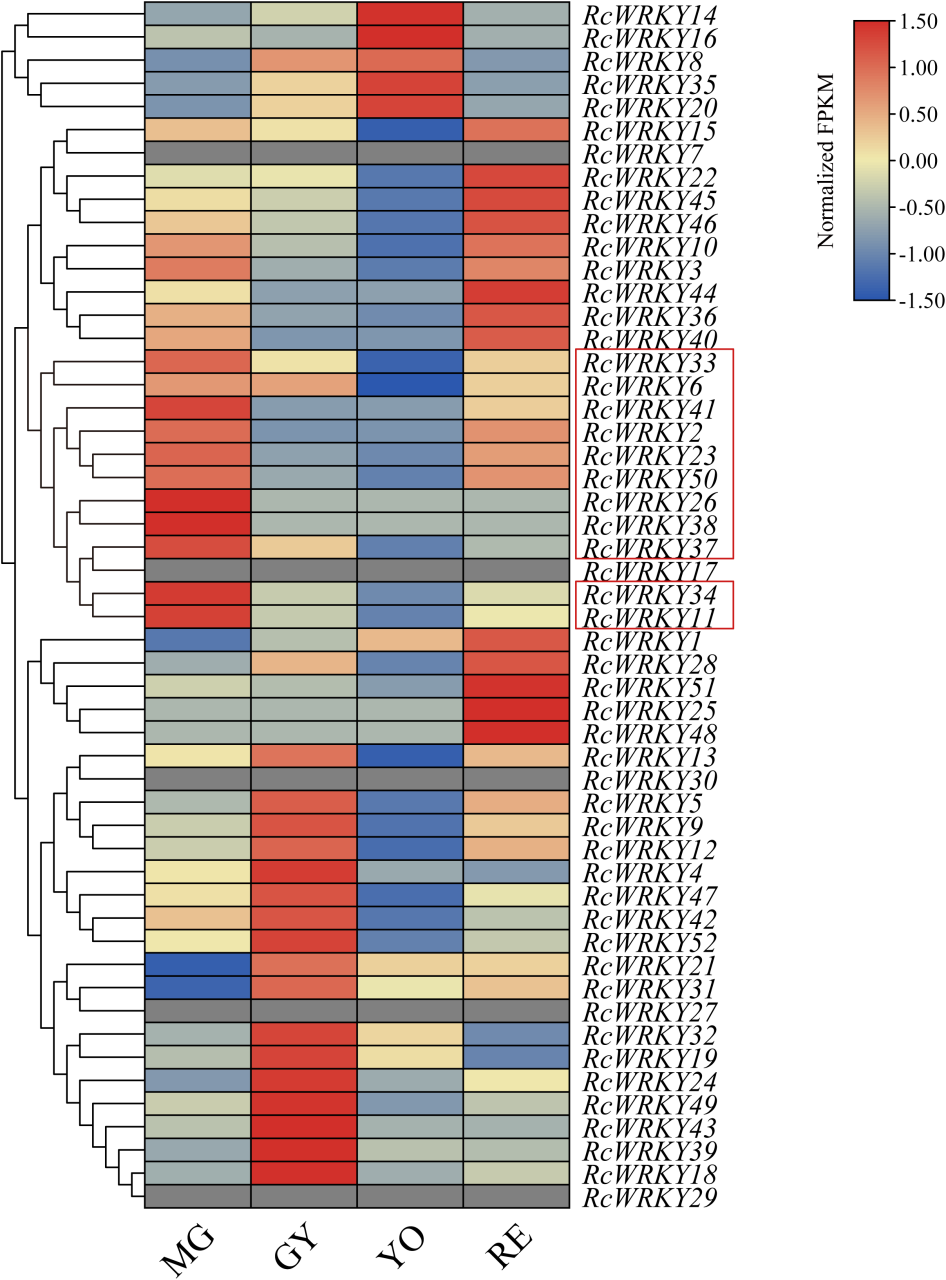
Expression patterns of the *RcWRKY* gene family during fruit ripening. mature green (MG), green yellow (GY),Yellow orange (YO), and Red (RE) during the growing season. Gray areas indicate that the gene was not expressed in the fruit. The genes enclosed in the red boxes were highly expressed during the MG period.

Transcriptome data analysis revealed that, among the 52 members, five were not expressed, whereas the other 47 members were highly expressed in fruits at different time points. Specifically, *RcWRKY33*, *RcWRKY6*, *RcWRKY41*, *RcWRKY2*, *RcWRKY23*, *RcWRKY50*, *RcWRKY26, RcWRKY38*, *RcWRKY37*, *RcWRKY34*, and *RcWRKY11* were highly expressed during the MG period, *RcWRKY13*, *RcWRKY5*, *RcWRKY9*, *RcWRKY12*, *RcWRKY4*, *RcWRKY47*, *RcWRKY42*, *RcWRKY52*, *RcWRKY21*, *RcWRKY31*, *RcWRKY32*, *RcWRKY19*, *RcWRKY24*, *RcWRKY49*, *RcWRKY43*, *RcWRKY39*, and *RcWRKY18* were highly expressed during the GY period, *RcWRKY14*, *RcWRKY16*, *RcWRKY8*, *RcWRKY35*, and *RcWRKY20* were highly expressed during the YO period, and *RcWRKY15*, *RcWRKY22*, *RcWRKY45*, *RcWRKY46*, *RcWRKY10*, *RcWRKY3*, *RcWRKY44*, *RcWRKY36*, *RcWRKY40*, *RcWRKY1*, *RcWRKY28*, *RcWRKY51*, *RcWRKY25*, and *RcWRKY48* were highly expressed during the RE period (**Figure 6**).

### 3.7 Expression patterns and correlation analysis of flavonoid synthesis-related gene families during fruit ripening

**Figure 7:**
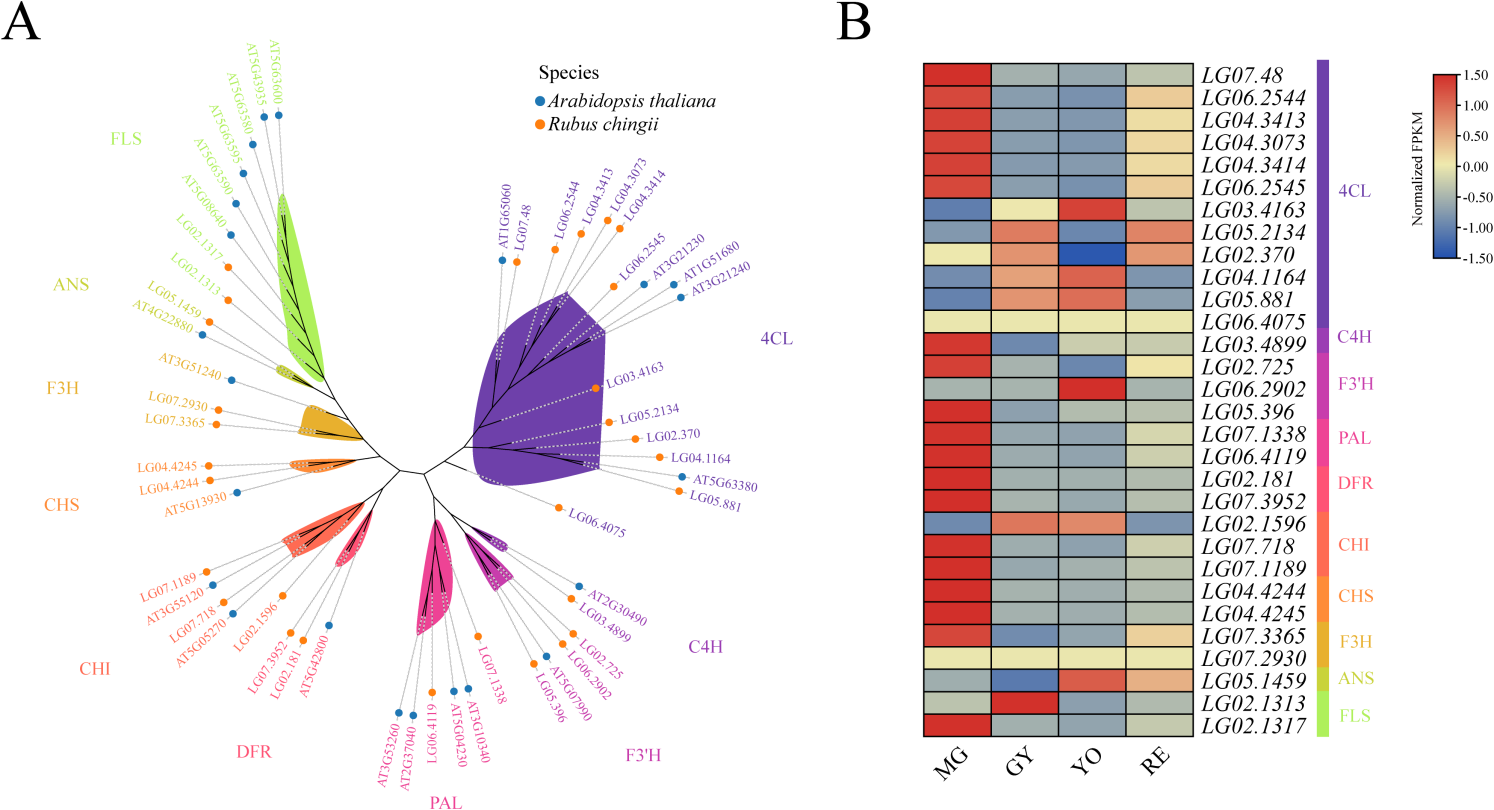
**(A)** Phylogenetic tree of the gene family related to flavonoid synthesis, **(B)** Expression pattern of the gene family related to flavonoid synthesis in *Rubus Chingii* during fruit ripening process.

In this study, we identified 30 flavonoid synthesis-related genes belonging to 10 gene families in *R. chingii*. These included 12 members of the *4CL* family, one member of the *C4H* family, three members of the *F3’H* family, two members of the *PAL* family, two members of the *DFR* family, three members of the *CHI* family, two members of the *CHS* family, two members of the *F3H* family, one member of the *ANS* family, and two members of the *FLS* family (**Figure 7A**). Further analysis of these 30 flavonoid synthesis-related genes in *R. chingii* revealed that most members exhibited highest expression levels during the MG period (**Figure 7B**).

**Figure 8:**
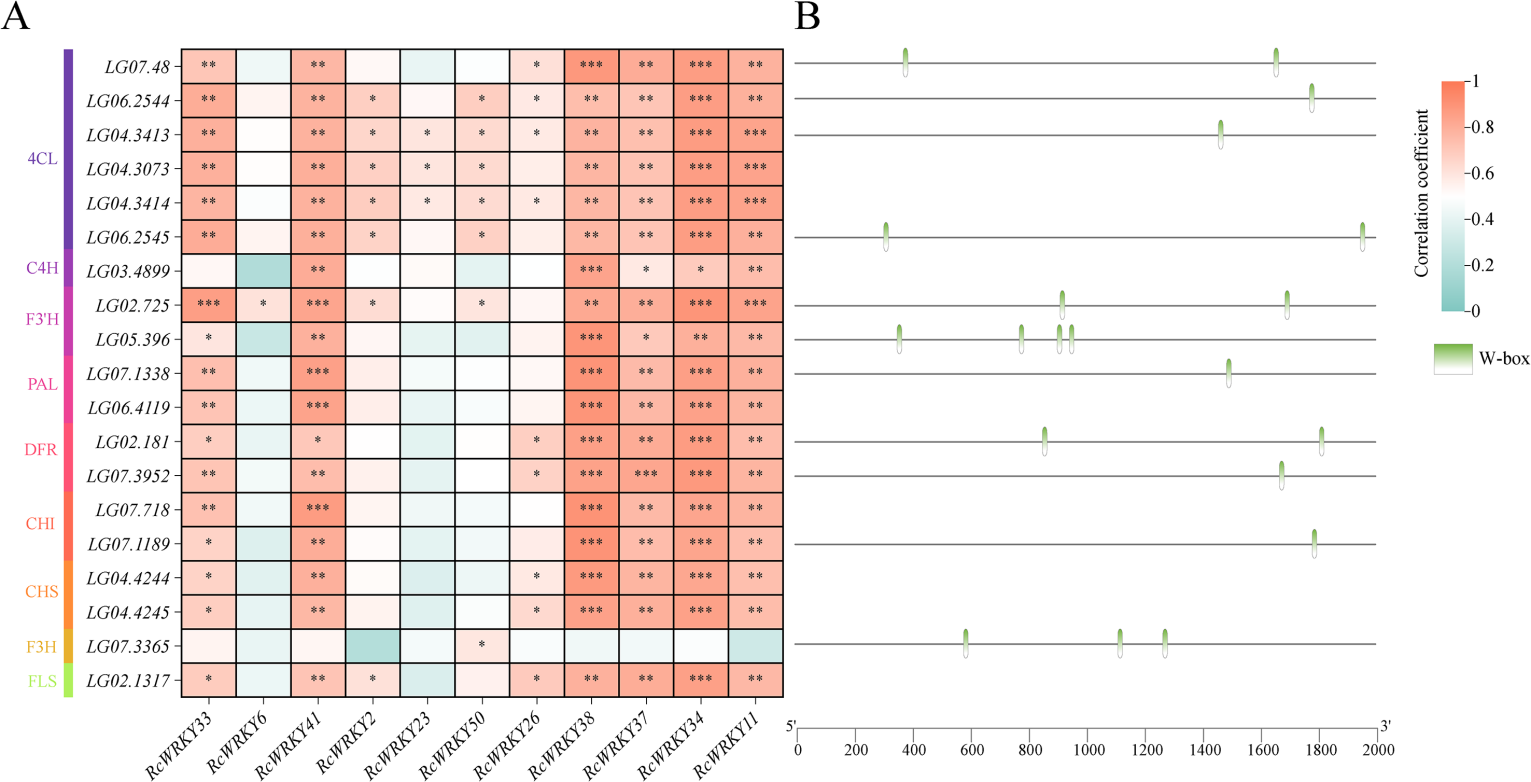
**(A)** Heat map of the correlation between the expression levels of flavonoid synthesis-related genes and *RcWRKY* genes in *Rubus Chingii*. **(B)** Expression patterns of flavonoid synthesis-related gene families in *Rubus Chingii* during fruit ripening. Stars represent the significance. One star represents P<0.05 in the student’s t-test, two stars represent P<0.01 in the student’s t-test, and three stars represent P<0.001 in the student’s t-test.

Correlation analysis was conducted between 19 flavonoid synthesis genes highly expressed during the MG period and 11 corresponding *WRKY* genes. Notably, *RcWRKY33*, *RcWRKY41*, *RcWRKY38*, *RcWRKY37*, *RcWRKY34*, and *RcWRKY11* were significantly positively correlated with most flavonoid synthesis genes (**Figure 8A**). Therefore, these six WAKY genes were subsequently selected for quantitative verification. Moreover, among the 19 flavonoid synthesis genes highly expressed during the MG period, 11 contained W-box *cis*-acting elements in their promoter regions (**Figure 8B**). From these 11 genes, the genes that contain more than two W-box elements and are significantly positively correlated with the 11 highly expressed waky genes during the same period were selected for subsequent quantitative verification and functional verification.

### 3.8 RT-qPCR, subcellular localization, and dual-luciferase reporter gene verification

**Figure 9:**
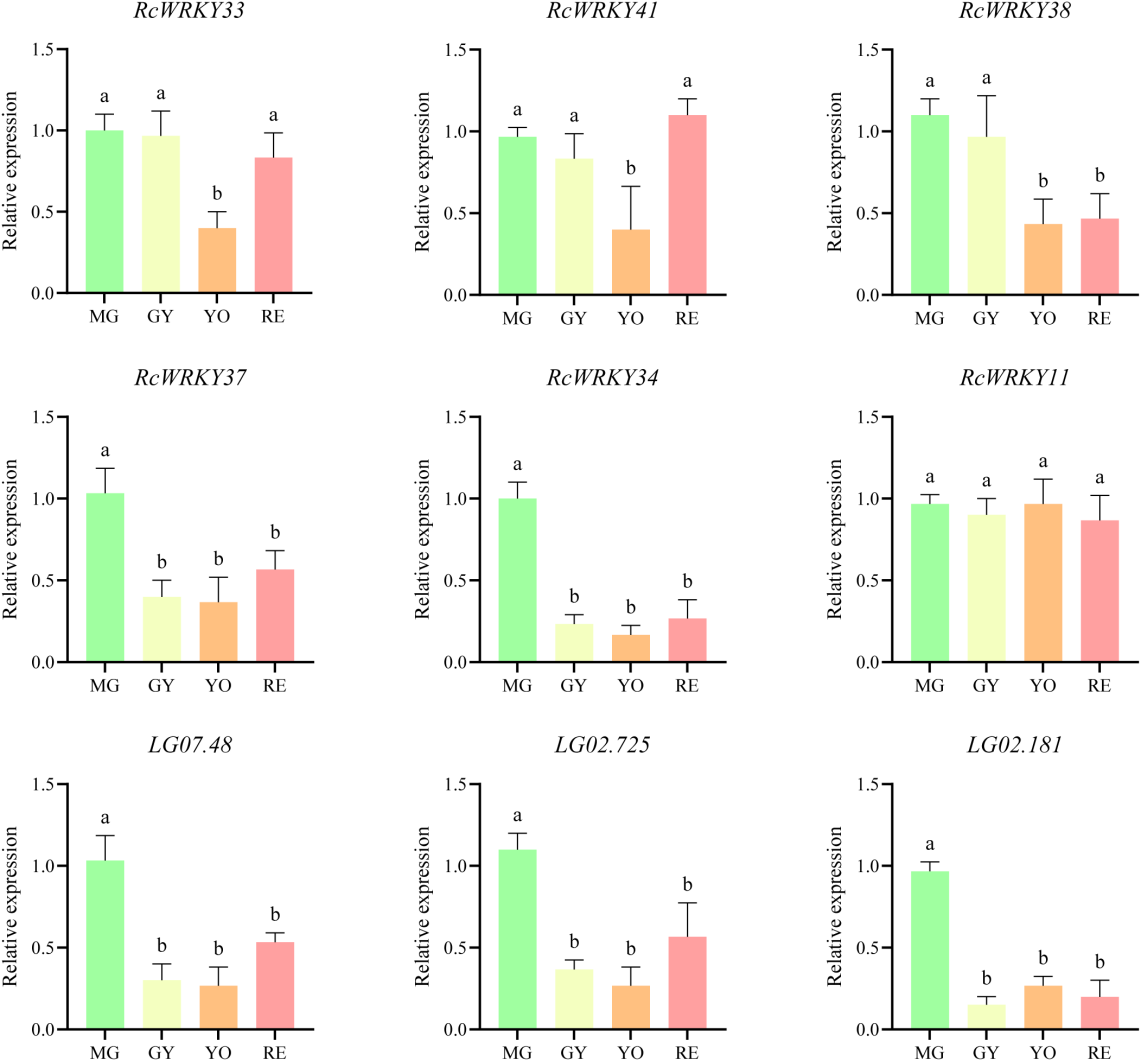
Relative expression levels of six *RcWRKY* gene family members and three genes related to flavonoid synthesis. The letters above the bars indicate significant differences, as determined by ANOVA test.

To verify the reliability of the transcriptome data, we conducted RT-qPCR validation of *RcWRKY33*, *RcWRKY41*, *RcWRKY38*, *RcWRKY37*, *RcWRKY34*, and *RcWRKY11*. *RcWRKY37* and *RcWRKY34* were highly expressed during the MG period (**Figure 9**). Simultaneously, we selected *LG07.48* from the *4CL* gene family, *LG02.725* from the F3’H gene family, and *LG02.181* from the *DFR* gene family for validation. All three genes were significantly highly expressed during the MG period (**Figure 9**).

**Figure 10:**
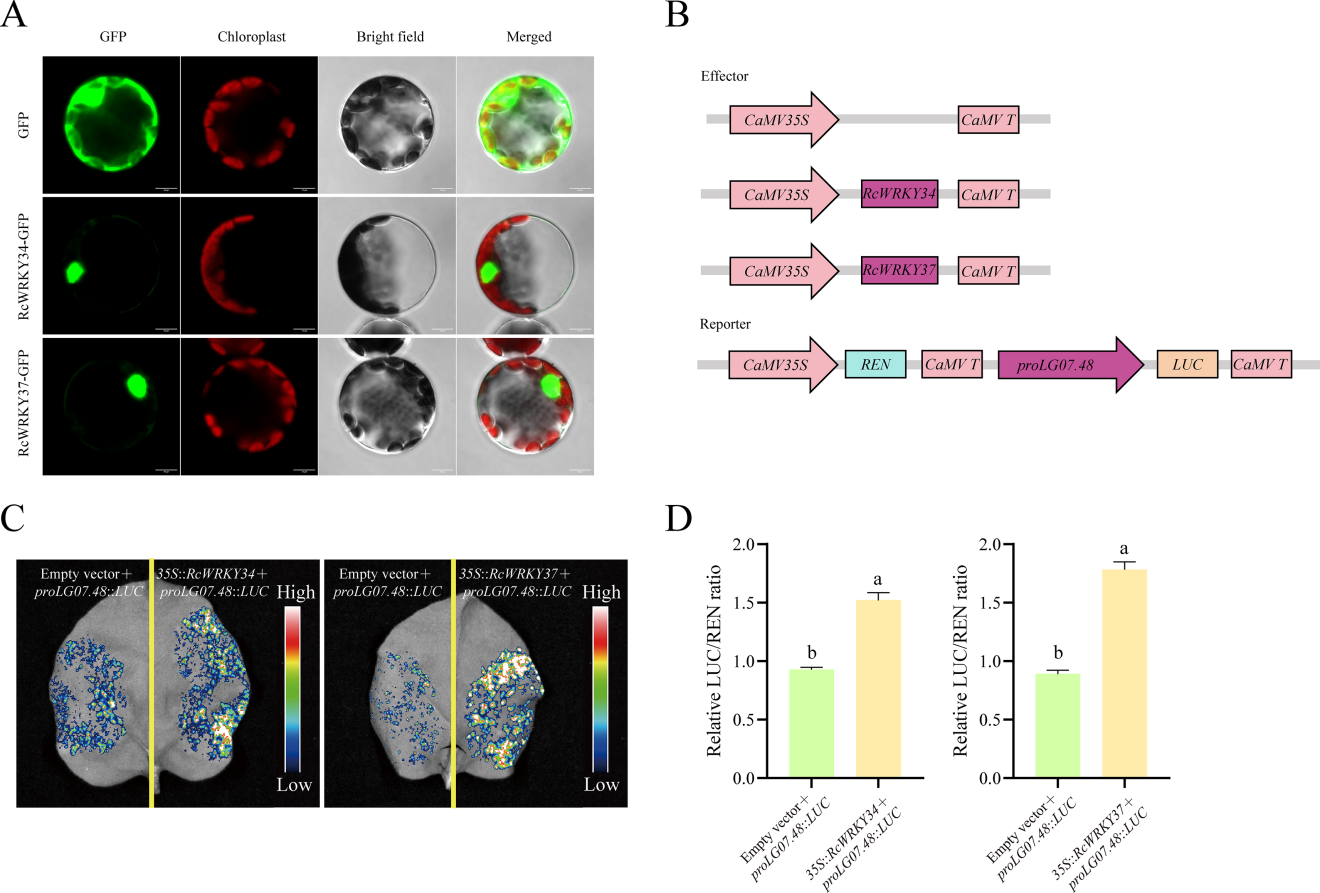
**(A)** Subcellular localization of *RcWRKY34* and *RcWRKY37*. **(B)** Constructs used for the LUC assay. **(C)** Luciferase activity intensity in leaves. **(D)** Relative LUC/REN fluorescence intensity. The letters above the bars indicate significant differences, as determined by the student’s t-test.

Subcellular localization studies confirmed that both *RcWRKY34* and *RcWRKY37* were located in the nucleus, consistent with the predicted results (**Figure 10A**). Based on **Figure 10B**, we constructed expression vectors and selected *RcWRKY34* and *RcWRKY37* as effector genes and *proLG07.48* as the reporter promoter to verify flavonoid synthesis regulation by the *RcWRKY* family in *R. chingii*. Fluorescence intensity was significantly higher in the tobacco leaves co-transfected with *35S::RcWRKY34* and *35S::RcWRKY37* than those transfected only with *proLG07.48::LUC* (**Figure 10C**). Quantitative analysis also confirmed higher luciferase activity in the leaves co-transfected with *35S::RcWRKY3*4 and *35S::RcWRKY37*, suggesting that both *RcWRKY34* and *RcWRKY37* specifically bind to *proLG07.48* and exert positive regulatory effects (**Figure 10D**).

## 4 Discussion

In this study, we identified 52 *RcWRKY* family members. Compared to the 89 *PsWRKY* family members identified in pea, 82 *CtWRKY* members in rose hip, 103 *CpWRKY* members in cucumber, 57 *BpWRKY* members in rockcress, and 55 *AhyWRKY* members in sorghum (Xiong et al., 2024; Song et al., 2023; Liu et al., 2024; Chen et al., 2025; Singh et al., 2025), only a few gene family members were identified in *R. chingii* in this study. However, consistent with previous reports, all members were predicted to be located in the nucleus. Construction of a phylogenetic tree revealed that, compared to those in various subgroups of *Arabidopsis* (Dong et al., 2024), RcWRKY family members, except group II-a members, were fewer in *R. chingii*. Systematic evolutionary analysis reveals that RcWRKY can be categorized into three main groups and seven subgroups, aligning closely with the classification of WRKY proteins in *Arabidopsis thaliana*. This alignment suggests that the core structure and function of WRKY proteins are conserved across plant species. However, motif analysis highlights the specific distribution of motifs within these subgroups, such as Motif 5 being exclusive to Group I, indicating a potential structural basis for functional differentiation among the various subgroups. Additionally, protein motifs of each subfamily were highly consistent with the gene structures, supporting the results of most previous studies on the *WRKY* family and confirming the conservation of the *WRKY* family in plants (Xiong et al., 2024; Song et al., 2023; Liu et al., 2024; Chen et al., 2025; Singh et al., 2025). Several *cis*-acting elements were identified in the promoters of *RcWRKY* family members, with light and jasmonic acid methyl response elements being the most common. Several *PsWRKY* family members in pea, *LrWRKY* members in lily, and *AsWRKY* members in myrtle respond to methyl jasmonate induction and exhibit changes in expression levels. These findings suggest that *RcWRKY* members also respond to methyl jasmonate (Xiong et al., 2024; Wu et al., 2005; Wang et al., 2023).

Gene duplication, the main mechanism to generate new genes and confer new biological functions, is crucial for plant evolution and adaptation to the environment (Xu et al., 2020). We identified 20 collinear gene pairs, The Ka/Ks values of the three gene pairs, RcWRKY1/RcWRKY35, RcWRKY9/RcWRKY36, and RcWRKY25/RcWRKY45, are all greater than 1 (or significantly close to/equal to 1), indicating that they are statistically “highly divergent” gene pairs, suggesting that they may have undergone positive selection or relaxed selection.17 of which exhibited Ka/Ks ratios < 1, indicating that *RcWRKY* underwent purifying selection. This is consistent with the *CtWRKY* family in rose hip, *CpWRKY* family in cucumber, *AhWRKY* family in peanut, and *LbWRKY* family in goji berry. Several studies have revealed that the *WRKY* family underwent purifying selection during evolution, suggesting the conservation of its function in plants (Song et al., 2023; Liu et al., 2024; Qu et al., 2023; Yan et al., 2022).One study found that WGD was important for *RcWRKY* generation, indicating that several *RcWRKY* members were generated from a single WGD event in *R. chingii* (Wu et al., 2025). TD had a significantly smaller impact on the expansion of *RcWRKY* than on that of *BjuWRKY*, *LaWRKY*, *CsWRKY*, and *TaWRKY* (Wang et al., 2021; Li et al., 2024; Kelimujiang et al., 2024; Ling et al., 2011). Previous studies have demonstrated the importance of WGD for plant adaptation to the environment (Ning et al., 2017), suggesting that *RcWRKY* members are closely associated with the environmental adaptation of *R. chingii*. Collinearity analysis of species helps to determine the time at which the members of a gene family are formed (Ren et al., 2022). In the *RcWRKY* family, only a few members showed collinearity with monocot plants, whereas most showed collinearity with dicot plants, indicating that most members were generated after the differentiation of monocot and dicot plants. Two members had no collinear genes, indicating that no additional *WRKY* genes were generated in *R. chingii* after formation. Generally, most *RcWRKY* family members exhibited collinearity, suggesting that their gene functions are similar to those in other species.

In the study, *RcWRKY33*, *RcWRKY6*, *RcWRKY41*, *RcWRKY2*, *RcWRKY23*, *RcWRKY50*, *RcWRKY26*, *RcWRKY38*, *RcWRKY37*, *RcWRKY34*, and *RcWRKY11* were highly expressed during the MG period. A previous study indicated that the total flavonoid content in *R. chingii* is highest during the MG period. Therefore, the identified 11 highly expressed *RcWRKY* genes during the MG period are possibly important for flavonoid synthesis in *R. chingii*. Subsequently, we identified 30 flavonoid synthesis-related genes from 10 gene families in *R. chingii*, most of which were highly expressed during the MG period, consistent with the high flavonoid content reported in *R. chingii* during this period. Correlation analysis revealed that *RcWRKY33*, *RcWRKY41*, *RcWRKY38*, *RcWRKY37*, *RcWRKY34*, and *RcWRKY11* were significantly positively correlated with most of the 19 flavonoid synthesis genes. Eleven of the 19 flavonoid synthesis genes contained W-box *cis*-acting elements, consistent with previous studies reporting several W-box *cis*-acting elements in the *4CL* gene family in *Scutellaria baicalensis* and *F3’H* and *DFR* families in walnut (Yang et al., 2025; Sha et al., 2025). To better verify the reliability of the transcriptome data, this study analyzed the relative expression levels of *RcWRKY33*, *RcWRKY41*, *RcWRKY38*, *RcWRKY37*, *RcWRKY34*, and *RcWRKY11* via RT-qPCR. Indeed, *RcWRKY37* and *RcWRKY34* were significantly highly expressed during the MG period. RT-qPCR also confirmed the expression of the *LG07.48* gene of the *4CL* family, *LG02.725* gene of the *F3’H* family, and *LG02.181* gene of the *DFR* family in *R. chingii*, consistent with the transcriptome data. These results align with previous reports that *CcWRKY25* in pepper exhibits the same expression patterns as *CcPAL* and *Cc4CL* and that *ZmWRKY82* in maize exhibits the same expression patterns as *ZmCHI6* (Zhang et al., 2023). *GhWRKY41* regulates *Gh4CL* expression to increase flavonoid content in cotton (Wang et al., 2025). *IiWRKY34* regulates *Ii4CL3* to enhance lignin synthesis in *Isatis indigotica* (Xiao et al., 2023). *PpWRKY70* regulates *Pp4CL* expression to increase flavonoid content in pear fruits (Xiao et al., 2020). Multiple *CchWRKY* genes regulate *Cch4CL1* in jujube (Xiao et al., 2023). Therefore, LUC test was used to assess whether *RcWRKY34* and *RcWRKY37* specifically bind to *proLG07.48* of the *4CL* family. Indeed, both *RcWRKY34* and *RcWRKY37* specifically bound to *proLG07.48* of the *4CL* family and exerted positive regulatory effects.

## 5 Conclusion

In this study, we identified 52 members of the *RcWRKY* gene family. The protein motifs, gene structures, replication types, etc. of the *RcWRKY* gene family were analyzed using bioinformatics, providing a comprehensive understanding of the members of the *RcWRKY* gene family. The collinearity analysis between species revealed that a large number of *RcWRKY* gene family members may have functions in other species as well, and that the *RcWRKY* gene family did not generate many new genes in *Rubus Chingii* formation. Through transcriptome analysis, 11 *RcWRKY* genes that were highly expressed during the MG period were identified, and a correlation analysis was conducted with 19 genes that were highly expressed in flavonoid synthesis. It was determined that 6 members may regulate flavonoid synthesis in *Rubus Chingii* fruit. Finally, RT-qPCR and LUC experiments verified that *RcWRKY37* and *RcWRKY34* were highly expressed during the MG period and could transcribe the flavonoid synthesis gene *proLG07.48*, exerting a positive regulatory effect. In the future, further in-depth exploration will be conducted on the mechanism by which *RcWRKY37* and *RcWRKY34* regulate flavonoid content in *Rubus Chingii* fruits.

## Data Availability

The datasets presented in this study can be found in online repositories. The names of the repository/repositories and accession number(s) can be found in the article/**Supplementary Material**.
