# Supplementary material for: Identification of the RcWRKY family in Rubus chingii and analysis of its regulatory mechanisms for flavonoid synthesis
Source: Front Plant Sci. 2026 Jan 29;16:1728584. doi: 10.3389/fpls.2025.1728584 (PMC12894398; doi:10.3389/fpls.2025.1728584)
Supplement: Supplementary file 1 [file Table1.docx]

Table S1:Domains of flavonoid gene family.

| Gene Family | Pfam ID | Domain Name |
| --- | --- | --- |
| *PAL* | PF00221 | Aromatic amino acid lyase |
| *4CL* | PF13193,PF00501 | AMP-binding enzyme C-terminal domain，AMP-binding enzyme domain |
| *CHS* | PF02797,PF00195 | Chalcone and stilbene synthases ,N/C-terminal domain |
| *CHI* | PF02431 | Chalcone-flavanone isomerase |
| *DFR* | PF01370 | NAD dependent epimerase domain |
| *F3H* | PF03171,PF14226 | 2OG-FeII_Oxy domain，DIOX_N domain |
| *FLS* |  |  |
| *ANS* |  |  |
| *C4H* | PF00067 | Cytochrome P450 |
| *F3'H* |  |  |

Table S2:Primer sequences for qRT-PCR.

| Gene | Primer ID | Primer Sequence (5'-3') |
| --- | --- | --- |
| *RcWRKY33* | RcWRKY33_F | TTGCTGTGTCCGTTGGGG |
| *RcWRKY33* | RcWRKY33_R | TGCCATGTGCCTCCAGTG |
| *RcWRKY41* | RcWRKY41_F | CCACAACCGTTGCAGCAG |
| *RcWRKY41* | RcWRKY41_R | ATGACATGGCGGGTGTGG |
| *RcWRKY38* | RcWRKY38_F | ACCTGAGGACCACGAGCA |
| *RcWRKY38* | RcWRKY38_R | TAAATGTGCCGGGCTGCA |
| *RcWRKY37* | RcWRKY37_F | GAGGTGCCCAGACGCAAT |
| *RcWRKY37* | RcWRKY37_R | CTTTTGCACGGTGTGGGC |
| *RcWRKY34* | RcWRKY34_F | ATGTCCGGCAACACTCCG |
| *RcWRKY34* | RcWRKY34_R | AATCCCCCTGATGCAGCG |
| *RcWRKY11* | RcWRKY11_F | CAGCAGCCCCAGTTCGAA |
| *RcWRKY11* | RcWRKY11_R | GCCACGTCTCCCCGTAAC |
| *LG07.48* | LG07.48_F | TCCCATGGTGGCGGACTA |
| *LG07.48* | LG07.48_R | GCCCAAGACTGCCTGAGG |
| *LG02.725* | LG02.725_F | TGGTCTCCTCGCCTTCGA |
| *LG02.725* | LG02.725_R | CGCCATAGGTCGCCGTAG |
| *LG02.181* | LG02.181_F | TGCAGCAACGTCGTCCAT |
| *LG02.181* | LG02.181_R | CACACAAACGGGTCCGGA |
| *Actin* | Actin_F | CGTCCTTTCCTTGTATGCTAGT |
| *Actin* | Actin_R | GGAGGGCATACCCTTCATAAAT |

Table S3:Protein Physicochemical Properties and Subcellular Localization Prediction.

| Gene name | Gene ID | Chromosome ID | Number of Amino Acid | Molecular Weight (Da) | Theoretical pI | Instability Index | Grand Average of Hydropathicity | Subcellular localization |
| --- | --- | --- | --- | --- | --- | --- | --- | --- |
| *RcWRKY1* | *LG02.35* | LG02 | 276 | 30985.58 | 8.85 | 43.7 | -0.811 | nucleus |
| *RcWRKY2* | *LG02.36* | LG02 | 359 | 39401.18 | 6.73 | 51.24 | -0.558 | nucleus |
| *RcWRKY3* | *LG02.456* | LG02 | 342 | 37258.28 | 9.38 | 54.22 | -0.488 | nucleus |
| *RcWRKY4* | *LG02.699* | LG02 | 323 | 35286.23 | 9.6 | 55.89 | -0.477 | nucleus |
| *RcWRKY5* | *LG02.914* | LG02 | 152 | 17567.04 | 5.24 | 46.51 | -1.189 | nucleus |
| *RcWRKY6* | *LG02.1691* | LG02 | 347 | 38844.88 | 9.64 | 57.46 | -0.775 | nucleus |
| *RcWRKY7* | *LG02.3461* | LG02 | 708 | 78566.73 | 5.42 | 44.28 | -0.354 | nucleus |
| *RcWRKY8* | *LG03.155* | LG03 | 573 | 62162.63 | 7.7 | 47.83 | -0.751 | nucleus |
| *RcWRKY9* | *LG03.694* | LG03 | 276 | 30417.55 | 5.14 | 58.84 | -0.791 | nucleus |
| *RcWRKY10* | *LG03.812* | LG03 | 348 | 38474.48 | 6.76 | 57.66 | -0.851 | nucleus |
| *RcWRKY11* | *LG03.1056* | LG03 | 801 | 87556.23 | 7.98 | 43.33 | -0.508 | nucleus |
| *RcWRKY12* | *LG03.2537* | LG03 | 1448 | 159535 | 6.56 | 52.93 | -0.838 | nucleus |
| *RcWRKY13* | *LG03.2662* | LG03 | 581 | 63111.59 | 6.5 | 52.4 | -0.795 | nucleus |
| *RcWRKY14* | *LG03.4827* | LG03 | 564 | 61536.67 | 6.71 | 58.47 | -1.01 | nucleus |
| *RcWRKY15* | *LG03.4971* | LG03 | 707 | 76266.45 | 5.73 | 48.41 | -0.8 | nucleus |
| *RcWRKY16* | *LG04.1250* | LG04 | 187 | 21348.86 | 9.43 | 43.03 | -0.906 | nucleus |
| *RcWRKY17* | *LG04.497* | LG04 | 612 | 66997.9 | 6.35 | 46.52 | -0.761 | nucleus |
| *RcWRKY18* | *LG04.597* | LG04 | 504 | 54820.87 | 6.29 | 55.43 | -0.961 | nucleus |
| *RcWRKY19* | *LG04.625* | LG04 | 278 | 29898.42 | 5.34 | 60.21 | -0.881 | nucleus |
| *RcWRKY20* | *LG04.1501* | LG04 | 223 | 24494.8 | 4.99 | 58.39 | -1.025 | nucleus |
| *RcWRKY21* | *LG04.3623* | LG04 | 299 | 32467.63 | 9.82 | 49.42 | -0.629 | nucleus |
| *RcWRKY22* | *LG05.281* | LG05 | 682 | 74006.19 | 6.43 | 47.26 | -0.855 | nucleus |
| *RcWRKY23* | *LG05.811* | LG05 | 158 | 17998.17 | 9.18 | 43.59 | -0.847 | nucleus |
| *RcWRKY24* | *LG05.1149* | LG05 | 278 | 31731.84 | 5.2 | 72.11 | -0.982 | nucleus |
| *RcWRKY25* | *LG05.1245* | LG05 | 305 | 34687.18 | 5.09 | 57.84 | -0.607 | nucleus |
| *RcWRKY26* | *LG05.1569* | LG05 | 312 | 35060.89 | 9.57 | 53.69 | -0.588 | nucleus |
| *RcWRKY27* | *LG05.3108* | LG05 | 544 | 60131.11 | 5.47 | 50.68 | -0.831 | nucleus |
| *RcWRKY28* | *LG05.4239* | LG05 | 273 | 31454.59 | 4.9 | 68.19 | -1.116 | nucleus |
| *RcWRKY29* | *LG06.452* | LG06 | 498 | 54136.64 | 6.65 | 52.63 | -0.578 | nucleus |
| *RcWRKY30* | *LG06.4720* | LG06 | 303 | 34594.46 | 5.78 | 45.35 | -0.677 | nucleus |
| *RcWRKY31* | *LG06.545* | LG06 | 526 | 57197.28 | 8.13 | 66.08 | -0.89 | nucleus |
| *RcWRKY32* | *LG06.996* | LG06 | 496 | 53338.02 | 5.57 | 47.22 | -0.628 | nucleus |
| *RcWRKY33* | *LG06.1116* | LG06 | 457 | 50357.16 | 8.62 | 39.71 | -0.778 | nucleus |
| *RcWRKY34* | *LG06.1385* | LG06 | 366 | 41448.64 | 6.62 | 57.11 | -1.01 | nucleus |
| *RcWRKY35* | *LG06.2466* | LG06 | 324 | 35782.11 | 8.72 | 46.71 | -0.755 | nucleus |
| *RcWRKY36* | *LG06.2579* | LG06 | 260 | 28455.66 | 5.05 | 55.77 | -0.693 | nucleus |
| *RcWRKY37* | *LG06.4214* | LG06 | 1228 | 133627.7 | 8.61 | 40.96 | -0.808 | nucleus |
| *RcWRKY38* | *LG06.4362* | LG06 | 288 | 32622.62 | 5.29 | 61.46 | -0.722 | nucleus |
| *RcWRKY39* | *LG06.4639* | LG06 | 462 | 51156.87 | 7.02 | 65.3 | -0.933 | nucleus |
| *RcWRKY40* | *LG06.4719* | LG06 | 327 | 36849.56 | 5.08 | 44.57 | -0.745 | nucleus |
| *RcWRKY41* | *LG06.5288* | LG06 | 309 | 34435.6 | 9.96 | 65.39 | -0.614 | nucleus |
| *RcWRKY42* | *LG06.5467* | LG06 | 686 | 75645.72 | 5.78 | 53.03 | -0.722 | nucleus |
| *RcWRKY43* | *LG07.250* | LG07 | 379 | 41678.46 | 6.06 | 53.47 | -0.598 | nucleus |
| *RcWRKY44* | *LG07.1640* | LG07 | 123 | 13520.87 | 4.78 | 33.37 | -0.692 | nucleus |
| *RcWRKY45* | *LG07.303* | LG07 | 422 | 45540 | 5.4 | 47.6 | -0.711 | nucleus |
| *RcWRKY46* | *LG07.698* | LG07 | 339 | 38005.93 | 5.46 | 43.77 | -0.827 | nucleus |
| *RcWRKY47* | *LG07.699* | LG07 | 249 | 27472.54 | 7.71 | 47.56 | -0.792 | nucleus |
| *RcWRKY48* | *LG07.1637* | LG07 | 425 | 49428.97 | 4.88 | 51.99 | -0.578 | nucleus |
| *RcWRKY49* | *LG07.2635* | LG07 | 208 | 23394.47 | 6.04 | 44.53 | -0.435 | nucleus |
| *RcWRKY50* | *LG07.3523* | LG07 | 426 | 46668.7 | 4.53 | 45.66 | -0.484 | nucleus |
| *RcWRKY51* | *LG07.3943* | LG07 | 338 | 37019.16 | 8.64 | 52.08 | -0.699 | nucleus |
| *RcWRKY52* | *LG07.4011* | LG07 | 353 | 39286.54 | 5.38 | 50.59 | -0.715 | nucleus |

Table S4:Motif Sequence.

| Motif ID | Sequence | Annotation |
| --- | --- | --- |
| Motif 1 | DIPDDGYSWRKYGQKPIKG | WRKY domain |
| Motif 2 | GCPARKQVZRSAEDPTILIT | WRKY domain |
| Motif 3 | SPYPRSYYRCT | Not found |
| Motif 4 | TYEGEHNHPLPASAT | Not found |
| Motif 5 | DGYNWRKYGQKQVKGSEYPRSYYKCTHPNCPVKKKVERSHDGQITEIIYK | WRKY domain |
| Motif 6 | ELEVLQAELGRVREENQRLKEMLTQVMEBYNALQMHLVDIM | WRKY domain |
| Motif 7 | KAVREPRVVVQTRSE | Not found |
| Motif 8 | LVAEATSAITADPNFTAALAAAITSIIGN | Not found |
| Motif 9 | CHCSKKRKSRVKRTIRVPAI | Zn-cluster domain |
| Motif 10 | DILDDGYRWRKYGQKVVKGNP | WRKY domain |

Table S5:The Ka, Ks, Ka/Ks Values of Gene Pairs Identified by McScanX.

| Gene pairs | | Ka | Ks | Ka/Ks |
| --- | --- | --- | --- | --- |
| *RcWRKY8* | *RcWRKY12* | 0.4089921507800397 | 2.323805644663824 | 0.17600101442184377 |
| *RcWRKY17* | *RcWRKY22* | 0.4635533655896907 | 2.133201202568542 | 0.2173040991311724 |
| *RcWRKY16* | *RcWRKY23* | 0.32257917471502146 | 2.2455049856898963 | 0.14365551480435215 |
| *RcWRKY13* | *RcWRKY33* | 0.5149848172459154 | 1.6275954283335308 | 0.3164083704592364 |
| *RcWRKY10* | *RcWRKY34* | 0.41732462993506136 | 2.1333027711055124 | 0.19562372279617718 |
| *RcWRKY1* | *RcWRKY35* | 0.59881490502892 | (NaN) Highly divergent gene sequences | |
| *RcWRKY9* | *RcWRKY36* | 0.3979324536290917 | (NaN) Highly divergent gene sequences | |
| *RcWRKY17* | *RcWRKY29* | 0.42416036100140364 | 2.2428827471796327 | 0.18911392560969778 |
| *RcWRKY22* | *RcWRKY29* | 0.4336228081090295 | 2.1109949521203633 | 0.20541157982091918 |
| *RcWRKY14* | *RcWRKY39* | 0.34278509236413734 | 1.5120009887184942 | 0.22670956892340854 |
| *RcWRKY6* | *RcWRKY41* | 0.304547422517429 | 1.204033186522355 | 0.25293939230783363 |
| *RcWRKY18* | *RcWRKY31* | 0.30455586948649144 | 1.5867921421181725 | 0.19193179837653265 |
| *RcWRKY8* | *RcWRKY32* | 0.2571857981718294 | 1.8742337258012527 | 0.13722183878741165 |
| *RcWRKY12* | *RcWRKY32* | 0.3248113024027691 | 2.1028241549527578 | 0.15446431963307286 |
| *RcWRKY25* | *RcWRKY45* | 0.5981268550088722 | (NaN) Highly divergent gene sequences | |
| *RcWRKY25* | *RcWRKY51* | 0.5261219873970602 | 2.525906403208829 | 0.20829037320175126 |
| *RcWRKY45* | *RcWRKY51* | 0.4692742537825171 | 2.7395505056081593 | 0.17129607679137923 |
| *RcWRKY24* | *RcWRKY52* | 0.38334190191163975 | 1.5827566058238691 | 0.2421988955857806 |
| *RcWRKY43* | *RcWRKY52* | 0.46324654680246014 | 1.7122351862515366 | 0.2705507692647117 |
| *RcWRKY40* | *RcWRKY46* | 0.565525038714336 | 2.2463777384502563 | 0.25174975207174355 |

Table S6:Duplication types of Gene pairs.

| Gene pairs | | Duplication types |
| --- | --- | --- |
| *RcWRKY8* | *RcWRKY12* | WGD |
| *RcWRKY17* | *RcWRKY22* | WGD |
| *RcWRKY16* | *RcWRKY23* | WGD |
| *RcWRKY13* | *RcWRKY33* | WGD |
| *RcWRKY10* | *RcWRKY34* | WGD |
| *RcWRKY1* | *RcWRKY35* | WGD |
| *RcWRKY9* | *RcWRKY36* | WGD |
| *RcWRKY17* | *RcWRKY29* | WGD |
| *RcWRKY22* | *RcWRKY29* | WGD |
| *RcWRKY14* | *RcWRKY39* | WGD |
| *RcWRKY6* | *RcWRKY41* | WGD |
| *RcWRKY18* | *RcWRKY31* | WGD |
| *RcWRKY12* | *RcWRKY32* | WGD |
| *RcWRKY8* | *RcWRKY32* | WGD |
| *RcWRKY25* | *RcWRKY45* | WGD |
| *RcWRKY25* | *RcWRKY51* | WGD |
| *RcWRKY45* | *RcWRKY51* | WGD |
| *RcWRKY24* | *RcWRKY52* | WGD |
| *RcWRKY43* | *RcWRKY52* | WGD |
| *RcWRKY40* | *RcWRKY46* | WGD |
| *RcWRKY26* | *RcWRKY3* | DSD |
| *RcWRKY3* | *RcWRKY4* | DSD |
| *RcWRKY28* | *RcWRKY11* | DSD |
| *RcWRKY3* | *RcWRKY21* | DSD |
| *RcWRKY4* | *RcWRKY21* | DSD |
| *RcWRKY4* | *RcWRKY26* | DSD |
| *RcWRKY21* | *RcWRKY26* | DSD |
| *RcWRKY15* | *RcWRKY42* | DSD |
| *RcWRKY1* | *RcWRKY2* | TD |
| *RcWRKY40* | *RcWRKY30* | TD |
| *RcWRKY20* | *RcWRKY34* | TRD |
| *RcWRKY27* | *RcWRKY29* | TRD |
| *RcWRKY49* | *RcWRKY43* | TRD |
